# Evaluation of Four Methods for Predicting Carbon Stocks of Korean Pine Plantations in Heilongjiang Province, China

**DOI:** 10.1371/journal.pone.0145017

**Published:** 2015-12-14

**Authors:** Huilin Gao, Lihu Dong, Fengri Li, Lianjun Zhang

**Affiliations:** 1 Department of Forest Management, School of Forestry, Northeast Forestry University, Harbin, Heilongjiang, People’s Republic of China; 2 Department of Forest and Natural Resources Management, College of Environmental Science and Forestry, State University of New York, Old Westbury, NY, United States of America; Chinese Academy of Forestry, CHINA

## Abstract

A total of 89 trees of Korean pine (*Pinus koraiensis*) were destructively sampled from the plantations in Heilongjiang Province, P.R. China. The sample trees were measured and calculated for the biomass and carbon stocks of tree components (i.e., stem, branch, foliage and root). Both compatible biomass and carbon stock models were developed with the total biomass and total carbon stocks as the constraints, respectively. Four methods were used to evaluate the carbon stocks of tree components. The first method predicted carbon stocks directly by the compatible carbon stocks models (Method 1). The other three methods indirectly predicted the carbon stocks in two steps: (1) estimating the biomass by the compatible biomass models, and (2) multiplying the estimated biomass by three different carbon conversion factors (i.e., carbon conversion factor 0.5 (Method 2), average carbon concentration of the sample trees (Method 3), and average carbon concentration of each tree component (Method 4)). The prediction errors of estimating the carbon stocks were compared and tested for the differences between the four methods. The results showed that the compatible biomass and carbon models with tree diameter (D) as the sole independent variable performed well so that Method 1 was the best method for predicting the carbon stocks of tree components and total. There were significant differences among the four methods for the carbon stock of stem. Method 2 produced the largest error, especially for stem and total. Methods 3 and Method 4 were slightly worse than Method 1, but the differences were not statistically significant. In practice, the indirect method using the mean carbon concentration of individual trees was sufficient to obtain accurate carbon stocks estimation if carbon stocks models are not available.

## Introduction

Carbon stocks estimation for the plot or the national level with reliable and verifiable techniques have always increased the social concerns [1–2], whereas the biomass calculation is the basis [3]. Compared to the popularized technique of remote sensing in biomass/carbon stocks monitoring and estimation, the traditional statistical technique which expensively and labour-intensively depends on the destructive sampling is still widely accepted due to high accurate estimation on biomass/carbon stocks at both tree and forest levels [4–6]. Allometric model always showed good performance for components (i.e., stem, branch, foliage and root) biomass prediction of individual trees or forest and carbon stocks will be converted by the estimated biomass combining with carbon conversion factor [7]. However, the accuracy of this method is in great demand to the accuracy of the carbon conversion factor for that some of the conversion factors were only empirical (e.g., 0.5) or simply conducted by species-specific of the trees [8]. As a result, incorporating the comparison of biomass model into the carbon concentration analysis is meaningful to the carbon stocks estimation.

A common practice for estimating tree carbon stocks is that tree biomass (total and components) models are first developed based on field-sampled data, and then carbon stock is calculated by multiplying the model-predicted biomass by a conversion factor of carbon concentration. The acceptable conversion factors include (1) the carbon concentration of 50% for woody tissues and 50% for non-woody tissues, or (2) 50% for woody and 45% for non-woody tissues [9–15]. In addition, carbon stock models were also established to directly predict carbon stocks of tree and components and total [16]. However, some noteworthy issues and problems still existed in these methods. Some reported biomass and carbon models may not satisfy the additivity or compatibility property among tree components and total equations. The impact of the prediction errors of tree biomass on estimating carbon stock by using conversion factors is lack of quantification.

Over the last decades, numerous allometric biomass models have been developed for many tree species around the world [17–21]. To ensure the additivity or compatibility of tree total and component biomass models, a variety of parameter estimation methods have been proposed [22–24]. Generalized method of moments (GMM) has been proven the efficiency in parameter estimation without the specification for the nature of the heteroscedasticity [25–27]. Maximum likelihood (ML) [23] and two-stage error-in-variable model (TSEM) [24] has been also employed and given the introductions in the researches [27]. However, seemingly unrelated regressions (SUR) [22, 25] and non-linear seemingly unrelated regressions (NSUR) [23] have been proven more general and flexible in recent years [28].

However, there have been limited efforts of developing tree carbon models, compared to biomass models, using tree attributes such as tree diameter and height [16]. Therefore, the carbon stocks of tree components and total are computed as the tree biomass predictions multiplied by carbon conversion factors, either an acceptable common constant (e.g., 0.5) or an empirical constant based on available data. Although this method worked well in some cases [16], there is lack of effort for quantitatively evaluating the error sources of estimating carbon stocks due to the biomass models and different carbon conversion factors, i.e., the prediction error through a sequence of operations on tree carbon stocks estimations. Furthermore, to our best knowledge, the additive system of biomass models performed well in estimating biomass and carbon stocks of total and tree components [29].

Korean pine (*Pinus koraiensis* Sieb. et Zucc) forests mainly distribute in Xiaoxing’an Mountain, and Changbai Mountain in Northeast of China. It is one of the important tree species for plantations in the area. In addition to the good quality of its lumber for industrial uses, its seeds are extensively harvested and sold as pine nuts, which have been the most widely traded pine nut in international commerce. The objectives of this study were (1) to develop additive systems of tree biomass and carbon stock models, (2) to evaluate four methods for estimating the carbon stocks of tree total and components, including a direct method (carbon stock models), and three indirect methods (i.e., 3 carbon conversion factors), (3) to compare and test the differences between the four methods.

## Data and Method

### Biomass and carbon stock data

**Fig 1:**
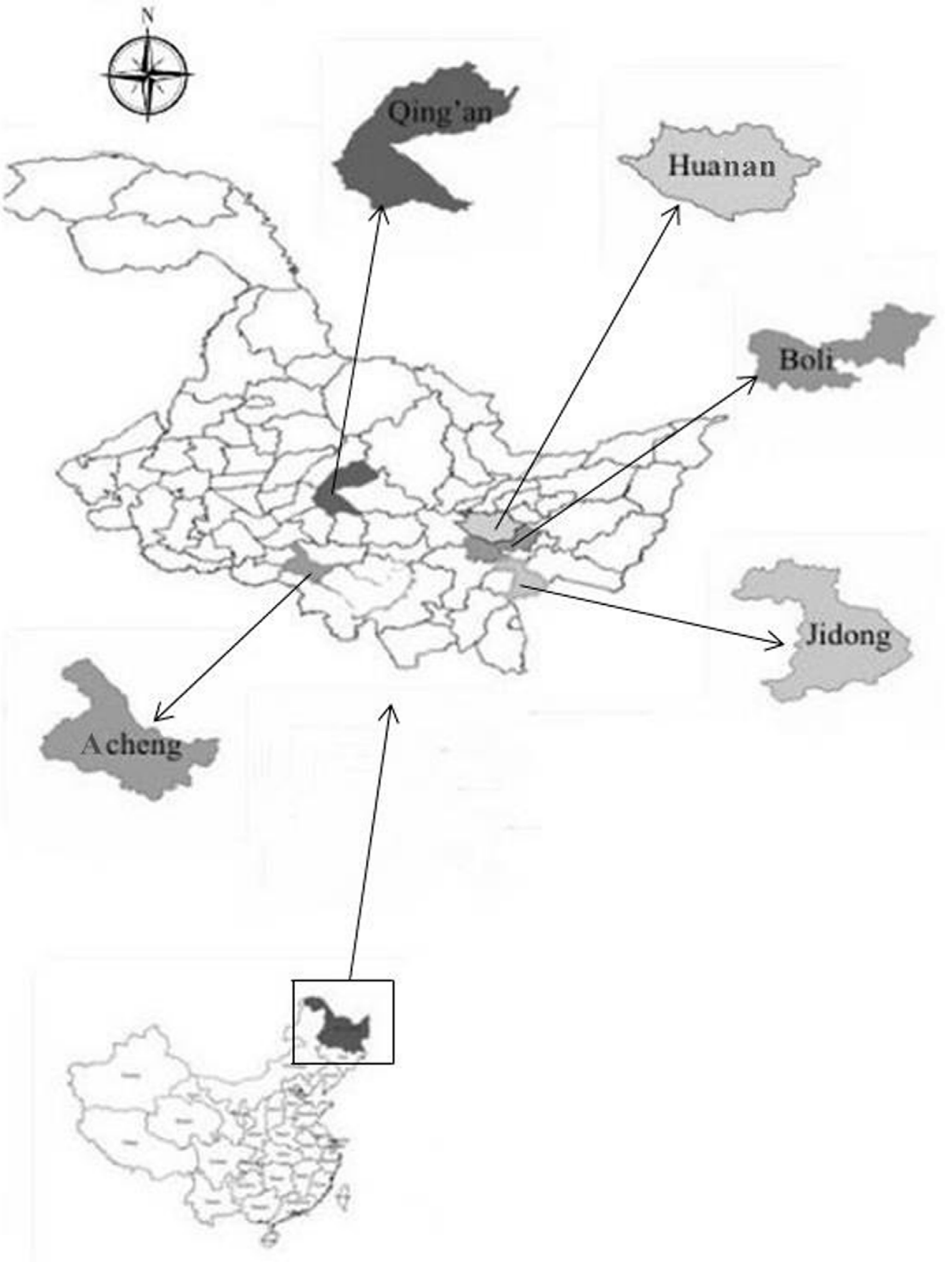
The geographical location of study area in the Heilongjiang province, China.

The study area is located in Heilongjiang province in Northeast China (43°25' − 53°33' N, 121°11' − 135°05' E) in which the forest area is 19.62 million hectares and the forest coverage is 43.16%. 5 counties were included in the study area (Fig 1) and the mean annual rainfall is 485 − 577mm and the mean annual temperature of 1.69 − 3.60°C. A total of 17 plots were selected in the different age of forest stand with a total of 89 Korean pine trees were destructively sampled in our study. The characteristics of the stand level for the study area were listed in Table 1. The data collection process in each site has been permitted by the local authority of each location. The names of the six official organizations were Mengjiagang forest farm, Boli forest bureau, Acheng forest bureau, Qing’an forest bureau and Jidong forest bureau. The whole process didn’t damage and disturb the natural environment. All the location of our study was carried out in the state-owned forest land not the private forest.

**Table 1:** Summary of the characteristics of the stand level for the study area.

Study area	Number of plots	Plot size (ha)	Age (year)	Density (trees ha^−1^)	Mean DBH (cm)	Slope (°)	Attitude (m)
**Huanan**	12	0.06–0.09	32–47	650–1650	15.5–22.0	< 5	194–263
**Boli**	2	0.27	35–44	2130–2135	12.6–12.7	12	382
**Acheng**	1	0.27	27	1470	12.7	0	269
**Qing’an**	1	0.27	32	804	17.4	< 5	228
**Jingdong**	1	0.27	37	1581	16.6	12	430

**Fig 2:**
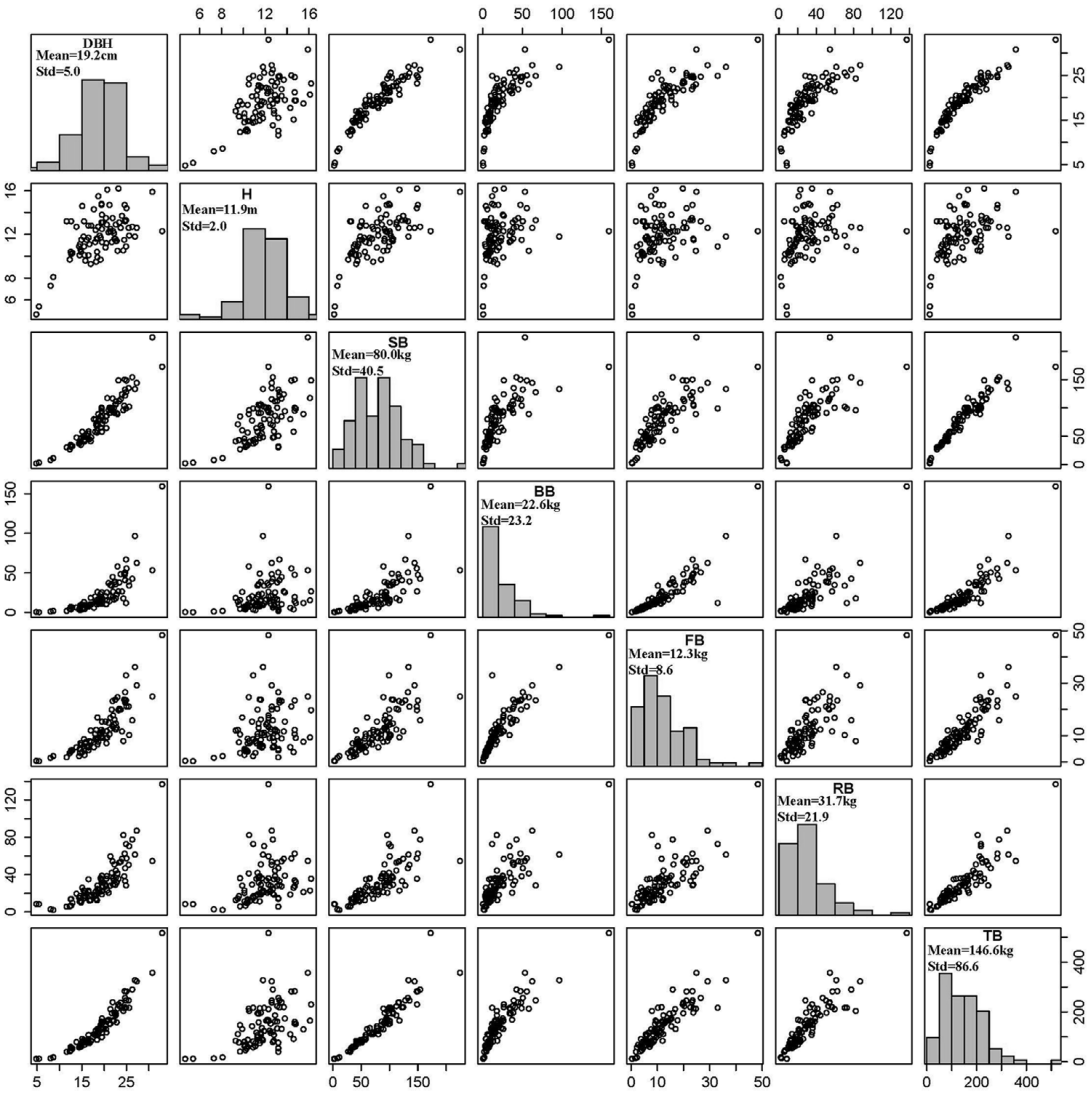
DBH (cm), total tree height (H, m) and components and total biomass (SB, BB, FB, RB and TB are stem, branch, foliage, root and total tree biomass in kg).

The sample trees were destructively felled at the ground level with tree diameter at breast height (D, cm), total tree height (H, m) and length of live crown (CL, m) measured and recorded in the field immediately. Each stem was cut into sections by the length of 1 m with each section weighed and recorded. Then, the live crowns were stratified into three layers along the stem. In each crown layer, 3 − 5 branches were selected as the samples and the branches and foliage were separated and weighed, respectively. We sampled about 50 − 100 g branches and foliage with the record of fresh weight and then took the samples to laboratory for moisture content determination. The tree roots were excavated using both a lifting machine and manual digging. The zone of excavating roots was limited to a circle of 3 m in radius, and the fine roots (< 5 mm) were not included. All roots of the sampled trees were divided into three categories: large roots (diameter ≥ 5 cm), medium roots (diameter 2 − 5 cm), and small roots (diameter < 2 cm). Each root class was sampled (about 100 − 200 g), weighed, and taken to laboratory for moisture content determination. All stem, branch, foliage, and root samples were oven-dried at 80°C and weighted. The dry biomass of each component was calculated by multiplying the fresh weight of each component by the dry / fresh ratio of each component. For each sample tree, the sum of branch dry biomass and foliage dry biomass yielded the crown dry biomass. The sum of crown dry biomass and stem dry biomass gave the aboveground biomass. The sum of aboveground dry biomass and root dry biomass produced the total tree biomass (Fig 2).

**Fig 3:**
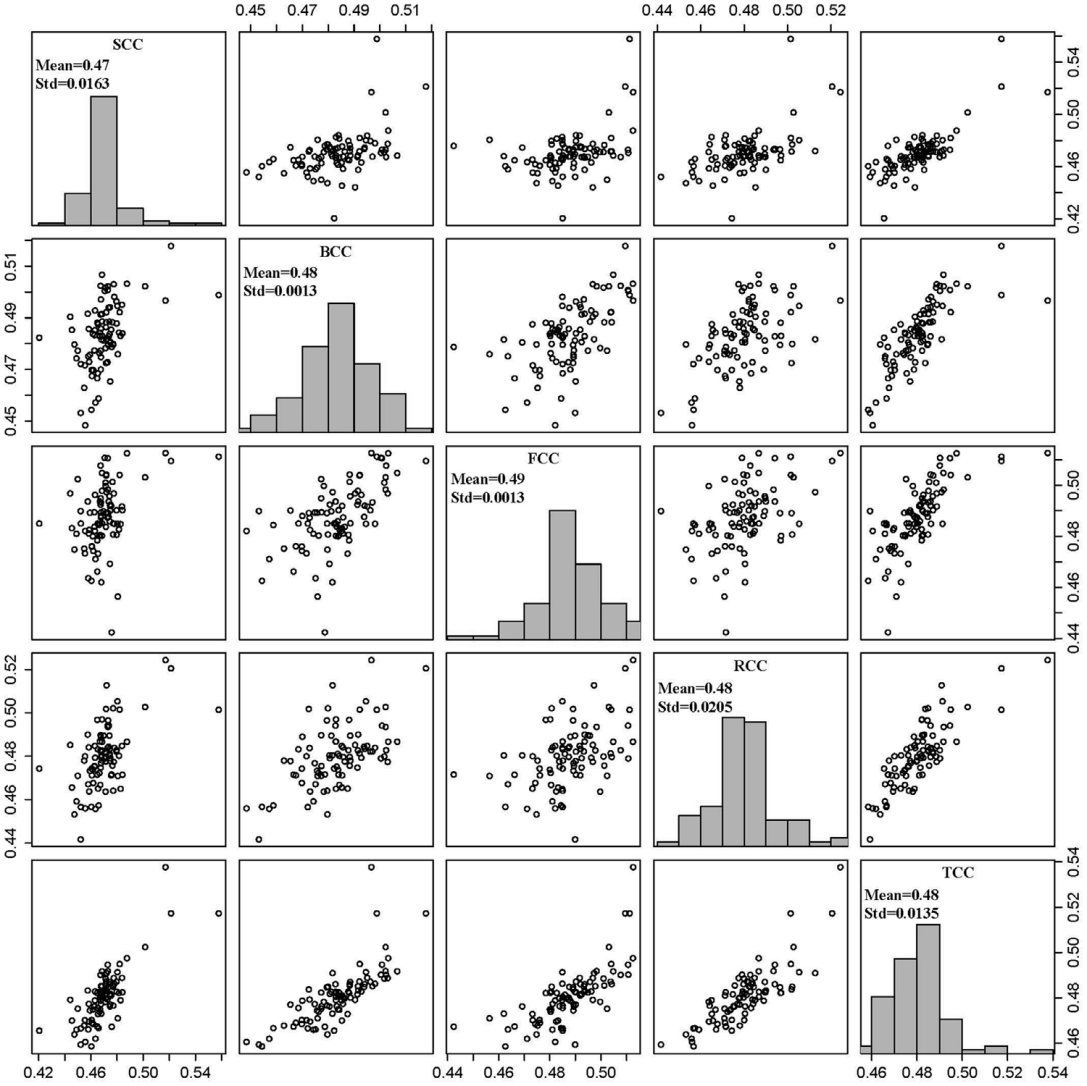
Tree components carbon concentration (SCC, BCC, FCC, RCC, TCC were stem, branch, foliage, root and total carbon concentration).

For each oven-dried sample of stem, branch, foliage and root approximately 50 mg was used for measuring carbon concentration using a Multi N/C 3000 analyzer with 1500 Solids Module (Analytik Jena AG, Germany). The samples were then burned completely at 1200°C in a vial containing pure oxygen, and the carbon emitted was measured with a non-dispersion infrared ray (NDIR) analyzer. The carbon stock of each component was then calculated by multiplying the biomass of each tree component by the respective carbon concentration. Thus, the carbon stock of individual tree was obtained by summing the component estimates. The carbon concentration for the each component and total tree were showed as Fig 3.

### Model specification and estimation

The data used to fit the models has been supported by the official authority and has no negative effect on the environment of human being and habitat of wildlife. Over the last two decades, researchers have tried different allometric equations for modeling tree biomass. The following equation was considered excellent in performance and remarkable in flexibility [22, 30, 31].

$$\text{Y}={\text{aX}}^{\text{b}}$$ (1)

where Y is response variable (tree biomass or carbon stocks), X is independent variable, a and b are model parameters to be estimated. Diameter at breast height which was defined to be 1.3m above the ground level, total tree height (H) and the combination forms were widely employed in the models. In our study, variable D and the combination form D^2^H were used to develop biomass and carbon stock models so that X can be D or D^2^H.

The additive systems of tree biomass and carbon stock models were formulated based on the methods by Tang and Li [32], in which the total tree biomass and carbon stocks were used as the constraints. The compatible biomass and carbon stock model which was derived from Eq 1 was showed as Eq 2.

$$\left\{ \begin{array}{l} {\text{Y}}_{\text{si}}={\text{a}}_{\text{i}}{\text{X}}^{{\text{b}}_{\text{i}}}\text{/(1}+{\text{r}}_{\text{1i}}{\text{X}}^{{\text{k}}_{\text{1i}}}+{\text{r}}_{\text{2i}}{\text{X}}^{{}^{{\text{k}}_{\text{2i}}}}+{\text{r}}_{\text{3i}}{\text{X}}^{{\text{k}}_{\text{3i}}}\text{)}\\ {\text{Y}}_{\text{bi}}={\text{a}}_{\text{i}}{\text{r}}_{\text{1i}}{\text{X}}^{{\text{b}}_{\text{i}}+{\text{k}}_{\text{1i}}}\text{/(1}+{\text{r}}_{\text{1i}}{\text{X}}^{{\text{k}}_{\text{1i}}}+{\text{r}}_{\text{2i}}{\text{X}}^{{}^{{\text{k}}_{\text{2i}}}}+{\text{r}}_{\text{3i}}{\text{X}}^{{\text{k}}_{\text{3i}}}\text{)}\\ {\text{Y}}_{\text{fi}}={\text{a}}_{\text{i}}{\text{r}}_{\text{2i}}{\text{X}}^{{\text{b}}_{\text{i}}+{\text{k}}_{\text{2i}}}\text{/(1}+{\text{r}}_{\text{1i}}{\text{X}}^{{\text{k}}_{\text{1i}}}+{\text{r}}_{\text{2i}}{\text{X}}^{{}^{{\text{k}}_{\text{2i}}}}+{\text{r}}_{\text{3i}}{\text{X}}^{{\text{k}}_{\text{3i}}}\text{)}\\ {\text{Y}}_{\text{ri}}={\text{a}}_{\text{i}}{\text{r}}_{\text{3i}}{\text{X}}^{{\text{b}}_{\text{i}}+{\text{k}}_{\text{3i}}}\text{/(1}+{\text{r}}_{\text{1i}}{\text{X}}^{{\text{k}}_{\text{1i}}}+{\text{r}}_{\text{2i}}{\text{X}}^{{}^{{\text{k}}_{\text{2i}}}}+{\text{r}}_{\text{3i}}{\text{X}}^{{\text{k}}_{\text{3i}}}\text{)} \end{array}\right.$$ (2)

where Y_si_, Y_bi_, Y_fi_, Y_ri_ are biomass or carbon stock for the stem, branch, foliage and root respectively. a_i_, b_i_, r_1i_, r_2i_, r_3i_, k_1i_, k_2i_ and k_3i_ are model parameters to be estimated using the jackknifing technique. The subscript i = 1, 2 were biomass models based on Y = aD^b^ and Y = a(D^2^H)^b^ which were defined as BM1, BM2 respectively and i = 3, 4 were carbon stock models based on Y = aD^b^ and Y = a(D^2^H)^b^ which were defined as CM3, CM4. The detailed derivation process for Eq 2 was showed in the Appendix.

Model [2] comprises a system of four nonlinear equations in our study. NSUR was applied to estimate the model coefficients in these nonlinear simultaneous equations using SAS/ETS PROC MODEL procedure [26]. NSUR employs the nonlinear joint generalized least squares to explain the correlations in the system of nonlinear biomass and carbon stock models [23, 25, 28]. Besides, NSUR also allow each model of the system to specify its own weighting function to overcome heteroscedasticity. To overcome the heteroscedasticity in biomass and carbon stock model, weighted functions were multiplied at the two side of the non-linear function with 1/D^x^ and (D^2^H)^p^ so that this problem can be resolved. The weighting function of each tree component for BM1 and CM3 was Y_i_ = resid.Y_i_/D^p^ (i = 1, 3 which has been defined above) and for BM2 and CM4 was Y_i_ = resid.Y_i_/(D^2^H)^p^. Error variance model e_i_ = x^p^ was developed (e_i_ is the model residual of the unweighted model, x is D or D^2^H) in which p was determined for each component [28].

### Model assessment and validation

In this study, jackknifing technique was used to assess the model parameters and validation model performance. The entire data size by leaving one out (n-1, n is the sample size) were used to estimate the model parameters, thus the one left sample not used in the model fitting was employed to predict by the fitted model. The R_a_^2^ (Eq 3) for each component and the total and the prediction for each left out independent variable were calculated for each round of fitting. The mean and standard deviation for each parameter were calculated. R_a_^2^ was used to assess the goodness-of-fit of the model. Due to the good performance in incorporating both variance of prediction error and the bias of prediction, the root mean squared error of prediction (RMSEp) was used as the model validation criteria [33, 34]. As a result, a total of five statistics (Eqs 5–9) were used to validate the model performance.

Adjusted coefficient of determination:

$${\text{R}}_{\text{a}}^{\text{2}}=\text{1}-\left(\text{1}-{\text{R}}^{\text{2}}\right)\left(\frac{\text{n}-\text{1}}{\text{n}-\text{p}-\text{1}}\right)$$ (3)

where

$${\text{R}}^{2}=1-\frac{\sum_{\text{i}=1}^{\text{n}}{\left({\text{Y}}_{\text{i}}-{\hat{\text{Y}}}_{\text{i,-i}}\right)}^{2}}{\sum_{\text{i}=1}^{\text{n}}{\left({\text{Y}}_{\text{i}}-\bar{\text{Y}}\right)}^{2}}$$ (4)

Root mean squared error of prediction:

$${\text{RMSE}}_{\text{p}}=\sqrt{\frac{\sum_{\text{i}=1}^{\text{n}}{\left({\text{Y}}_{\text{i}}-{\hat{\text{Y}}}_{\text{i,-i}}\right)}^{2}}{\text{n}-\text{p}}}$$ (5)

Mean prediction error:

$$\text{ME}=\frac{\text{1}}{\text{n}}\sum_{\text{i}=\text{1}}^{\text{n}}{\text{(Y}}_{\text{i}}-{\hat{\text{Y}}}_{\text{i, -i}}\text{)}$$ (6)

Mean prediction error percent:

$$\text{ME\%}=\frac{\text{1}}{\text{n}}\sum_{\text{i}=\text{1}}^{\text{n}}\left(\frac{{\text{Y}}_{\text{i}}-{\hat{\text{Y}}}_{\text{i, -i}}}{{\text{Y}}_{\text{i}}}\right)\times \text{100}$$ (7)

Mean absolute error:

$$\text{MAE}=\sum_{\text{i}=\text{1}}^{\text{n}}\left|\frac{{\text{Y}}_{\text{i}}-{\hat{\text{Y}}}_{\text{i,- i}}}{\text{n}}\right|$$ (8)

Mean absolute percent error:

$$\text{ MAE\%}=\frac{\text{1}}{\text{n}}\sum_{\text{i}=\text{1}}^{\text{n}}\left|\frac{{\text{Y}}_{\text{i}}-{\hat{\text{Y}}}_{\text{i, -i}}}{{\text{Y}}_{\text{i}}}\right|\times \text{100}$$ (9)

Where Y_i_ is the *i*th observed value of response variable (i.e., biomass or carbon stock), ${\hat{\text{Y}}}_{\text{i,}-\text{i}}$ is the *i*th predicted value for the dependent variable without inclusion in the fitting dataset, **$\bar{\text{Y}}$** is the mean of observed response variable, n is sample size, and p is the number of model parameters.

### Comparison of carbon stock estimation by four methods

Four methods were used in this study to evaluate the carbon stocks of tree components and total: (1) the developed additive systems of carbon models (CM3 and CM4) were directly used to compute the carbon stocks of tree components and total, given tree D and H (Method 1); (2) the developed additive systems of biomass models (BM1 and BM2) were first used to estimate the biomass of tree components and total, and then the predicted biomass was multiplied by the carbon conversion factor 0.5 (Method 2); (3) the developed additive systems of biomass models were first used to estimate the biomass of tree components and total, and then the predicted biomass was multiplied by the average carbon concentration of total tree (i.e., 0.48; Fig 3) (Method 3); and (Eq 4) the developed additive systems of biomass models were first used to estimate the biomass of tree components and total, and then the predicted biomass was multiplied by the average carbon concentration of each tree component (i.e., stem 0.47, branch 0.48, foliage 0.49, and root 0.48; Fig 3) (Method 4). The last three methods were considered as the indirect methods.

Relative root mean squared error (RMSE_r_) (Eq 10) was used to compare the prediction error of the four methods. Thus, the relative root mean squared error for carbon (RMSE_rc_) was calculated and RMSE_rc_ of indirect method was based on Eq 11.

$${\text{RMSE}}_{\text{r}}=\sqrt{\frac{\sum_{\text{i}=\text{1}}^{\text{n}}{\left({\text{Y}}_{\text{i}}-{\hat{\text{Y}}}_{\text{i, -1}}\right)}^{\text{2}}}{\text{n}-\text{p}}}\cdot \frac{\text{1}}{\hat{\bar{\text{Y}}}}\times 100$$ (10)

RMSErc=∑i=1n(Cmi−C^mi, -mi)2n−p⋅1C¯^m×100 (11)

where C_mi_ is the *i*th observed value of carbon stock for the *m* tree components (*m* = 1, 2, 3, and 4 denotes stem, branch, foliage and root, respectively), ${\hat{\text{C}}}_{\text{mi, -mi}}$ is the *i*th predicted value of carbon stock without inclusion in the fitting dataset for the *m* tree components. ${\hat{\bar{\text{C}}}}_{\text{m}}$ is the mean of predicted value of carbon stock for the *m* tree components with the 89 rounding processes, and the other symbols were defined as above.

Given Eq 11, RMSE_rc_ of indirect method was derived as Eqs 12 and 13.

$${\text{RMSE}}_{\text{rc}}=\frac{\text{1}}{\sqrt{\text{n}-\text{p}}}\cdot \frac{{\sqrt{\sum_{\text{i}=\text{1}}^{\text{n}}{\left({\text{W}}_{\text{mi}}\cdot {\text{C}}_{\text{mi}}\text{\%}-\overset{}{{\hat{\text{W}}}_{\text{mi, -mi}}\cdot }{\bar{\text{C}}}_{\text{mk}}\text{\%}\right)}^{\text{2}}}}^{}}{\frac{\text{1}}{\text{n}}\cdot \sum_{\text{i}=\text{1}}^{\text{n}}{\hat{\text{W}}}_{\text{mi, -mi}}\cdot \overset{}{{\bar{\text{C}}}_{\text{mk}}\text{\%}}}\times 100$$ (12)

$${\text{RMSE}}_{\text{rc}}=\frac{\text{1}}{\sqrt{\text{n}-\text{p}}}\cdot \sqrt{\sum_{\text{i}=\text{1}}^{\text{n}}{\left({\text{W}}_{\text{mi}}\cdot \frac{{\text{C}}_{\text{m}}{}_{\text{i}}\text{\%}}{{\overset{\_\_}{\text{C}}}_{\text{mk}}\text{\%}}-{\hat{\text{W}}}_{\text{mi, -mi}}\right)}^{\text{2}}}\cdot \frac{\text{1}}{{\hat{\bar{\text{W}}}}_{\text{m}}}\times 100$$ (13)

Where W_mi_ is the *i*th observed value of biomass for the *m* tree components (*m* = 1, 2, 3, and 4 denotes stem, branch, foliage and root, respectively), ${\hat{\text{W}}}_{\text{mi, -mi}}$ is the *i*th predicted value of biomass without inclusion in the fitting dataset for the *m* tree components, ${\hat{\bar{\text{W}}}}_{\text{m}}$ is the mean of predicted value of biomass of the *m* tree components with the 89 rounding processes, C_mi_% is the *i*th observed value of carbon concentration for the *m* tree components, ${\bar{\text{C}}}_{\text{mk}}\text{\%}$ is the *k*th carbon conversion factor used in the three indirect methods for the *m* tree components (*k* = 1 denotes carbon conversion factor 0.5, *k* = 2 denotes the average carbon concentration of total tree 0.48, and *k* = 3 denotes the average carbon concentration of each tree component).

To test whether the significant difference will be produced by the four methods for predicting carbon stocks of the tree components and total, the analysis of variance with randomized completed block design (RCBD) was used [35–37], in which the treatment was the four methods and blocking was each tree because the four methods were applied to each tree simultaneously. The least significant difference (LSD) was used for multiple mean comparisons. The PROC GLM procedure of SAS 9.3 was used for computation [38, 39], and the significance level was set at α = 0.05.

## Results

### Model fitting and validation

The mean values and standard deviations of the each parameter for the compatible biomass models (i.e., BM1 and BM2) and the carbon stock models (i.e., CM3 and CM4) using jackknifing technique were listed in Table 2. The mean values and standard deviation of R_a_^2^ for compatible biomass and carbon stock models were listed in Table 3. Validation results based on jackknifing technique for compatible biomass models were showed in Table 4 and for compatible carbon stock models were showed in Table 5. The results indicated that the mean of R_a_^2^ of all the components and total for BM1 and BM2 had R_a_^2^ ≥ 0.75, and R_a_^2^ ≥ 0.73, respectively, while the jackknifing validation results were RMSE_p_ ≤ 22.10 kg, ME ≤ ±0.28 kg (ME% ≤ ± 7.24%), MAE≤ 12.89 kg (MAE ≤ 28.01%) for all components and total of BM1 and RMSE_p_ ≤ 23.89 kg, ME ≤ ±0.31 kg (ME% ≤ ±8.19%), MAE≤ 13.67 kg (MAE ≤ 31.01%) for BM2, respectively. Similarly, the R_a_^2^ of tree components and total for CM3 and CM4 had R_a_^2^ ≥ 0.74, and R_a_^2^ ≥ 0.74, respectively, while the validation results were RMSE_p_ ≤ 10.59 kg, ME ≤ ±1.21 kg (ME% ≤ ± 13.99%), MAE≤ 6.54 kg (MAE ≤ 28.49%) for CM3 and RMSE_p_ ≤ 10.66 kg, ME ≤ ±1.32 kg (ME% ≤ ±16.14%), MAE≤ 6.89 kg (MAE ≤ 30.10%) for CM4 respectively.

**Table 2:** The mean and standard deviation of the parameter for biomass and carbon stock models.

Model parameter	BM1		BM2		CM3		CM4	
	Mean	Std	Mean	Std	Mean	Std	Mean	Std
**a**_**i**_	0.0891	0.0042	0.0374	0.0042	0.0451	0.0009	0.0201	0.0034
**b**_**i**_	2.4560	0.0128	2.5120	0.0201	2.5001	0.0108	0.9814	0.0204
**r**_**1i**_	0.0009	0.0011	0.0751	0.0012	0.0043	0.0012	0.1021	0.0075
**r**_**2i**_	0.0240	0.0025	0.2210	0.0032	0.0302	0.0082	0.4441	0.0037
**r**_**3i**_	0.0892	0.0141	0.0848	0.0142	0.0714	0.0212	0.1541	0.0850
**k**_**1i**_	1.8254	0.0014	0.4701	0.0021	1.2801	0.0310	0.1021	0.0454
**k**_**2i**_	0.6090	0.0347	-0.1302	0.0420	0.5205	0.0296	-0.1213	0.0421
**k**_**3i**_	0.4901	0.0424	0.4924	0.0801	0.5713	0.0585	0.1091	0.0577

The weighting factors were D^2.33^, D^2.01^, D^1.67^, D^2.19^ for BM1, (D^2^H)^0.77^, (D^2^H)^0.82^, (D^2^H)^0.60^, (D^2^H)^0.75^ for BM2, D^2.22^, D^2.13^, D^1.93^, D^2.28^ for CM3 and (D^2^H)^0.60^, (D^2^H)^0.67^, (D^2^H)^0.72^, (D^2^H)^0.81^ for CM4 for stem, branch, foliage and root respectively.

**Table 3:** The mean and standard deviation of R_a_^2^ for biomass and carbon stock models.

Models	Statistics	Stem	Branch	Foliage	Root	Total
**BM1**	Mean	0.88	0.78	0.79	0.75	0.90
	Std	0.0021	0.0112	0.0074	0.0079	0.0019
**BM2**	Mean	0.91	0.73	0.76	0.81	0.89
	Std	0.0013	0.0114	0.0061	0.0094	0.0069
**CM3**	Mean	0.91	0.77	0.76	0.74	0.89
	Std	0.0011	0.0062	0.0051	0.0041	0.0010
**CM4**	Mean	0.93	0.75	0.74	0.77	0.88
	Std	0.0011	0.0058	0.0049	0.0047	0.0008

**Table 4:** Jackknifing validation for compatible biomass models.

Model	Component	RMSE_p_(kg)	ME(kg)	ME%	MAE(kg)	MAE%
**BM1**	Stem	15.09	-0.21	-3.61	9.98	13.38
	Branch	13.15	0.28	7.24	7.51	18.58
	Foliage	4.39	0.06	7.17	2.91	28.01
	Root	11.68	-0.13	-7.13	8.09	27.98
	Total	22.10	0.08	3.14	12.89	10.94
**BM2**	Stem	14.13	0.09	1.23	8.69	11.49
	Branch	13.38	0.31	7.61	7.98	19.25
	Foliage	4.87	0.31	8.19	4.09	31.01
	Root	10.01	-0.20	-6.41	7.31	24.32
	Total	23.89	0.11	3.45	13.67	11.08

**Table 5:** Jackknifing validation for compatible carbon stock models.

Model	Component	RMSE_p_(kg)	ME(kg)	ME%	MAE(kg)	MAE%
**CM3**	Stem	6.38	-0.18	-3.18	4.33	13.10
	Branch	5.29	-0.05	-13.99	3.31	26.99
	Foliage	2.20	0.02	8.63	1.51	18.66
	Root	7.08	0.11	6.89	4.12	28.49
	Total	10.59	1.21	4.30	6.54	16.19
**CM4**	Stem	5.15	0.11	1.24	2.70	8.65
	Branch	5.38	-0.08	-16.14	3.51	30.10
	Foliage	2.65	-0.03	-9.20	1.63	18.75
	Root	6.48	0.09	6.30	4.10	28.05
	Total	10.66	1.32	4.57	6.89	17.15

It was evident that BM2 fitted the data better (higher R_a_^2^) than did BM1 for stem and root, indicating that adding tree H into the biomass model increased the explanation power of these two components. The jackknifing validation also proved this conclusion with significant larger RMSE_p_, ME and MAE for stem and root for BM1 compared to BM2. However, BM2 was much inferior to BM1 when considering the branch, foliage and the total. For CM3 and CM4, the comparison results explained the similar conclusion to BM1 and BM2. However, BM2 and CM4 have much model parameters to be estimated and the correlations among these parameters could not be ignored. Thus, we preferred to specify the compatible biomass and carbon stock models using D as the only independent variable to make methods comparison.

### Comparison of four methods in carbon stock prediction

**Fig 4:**
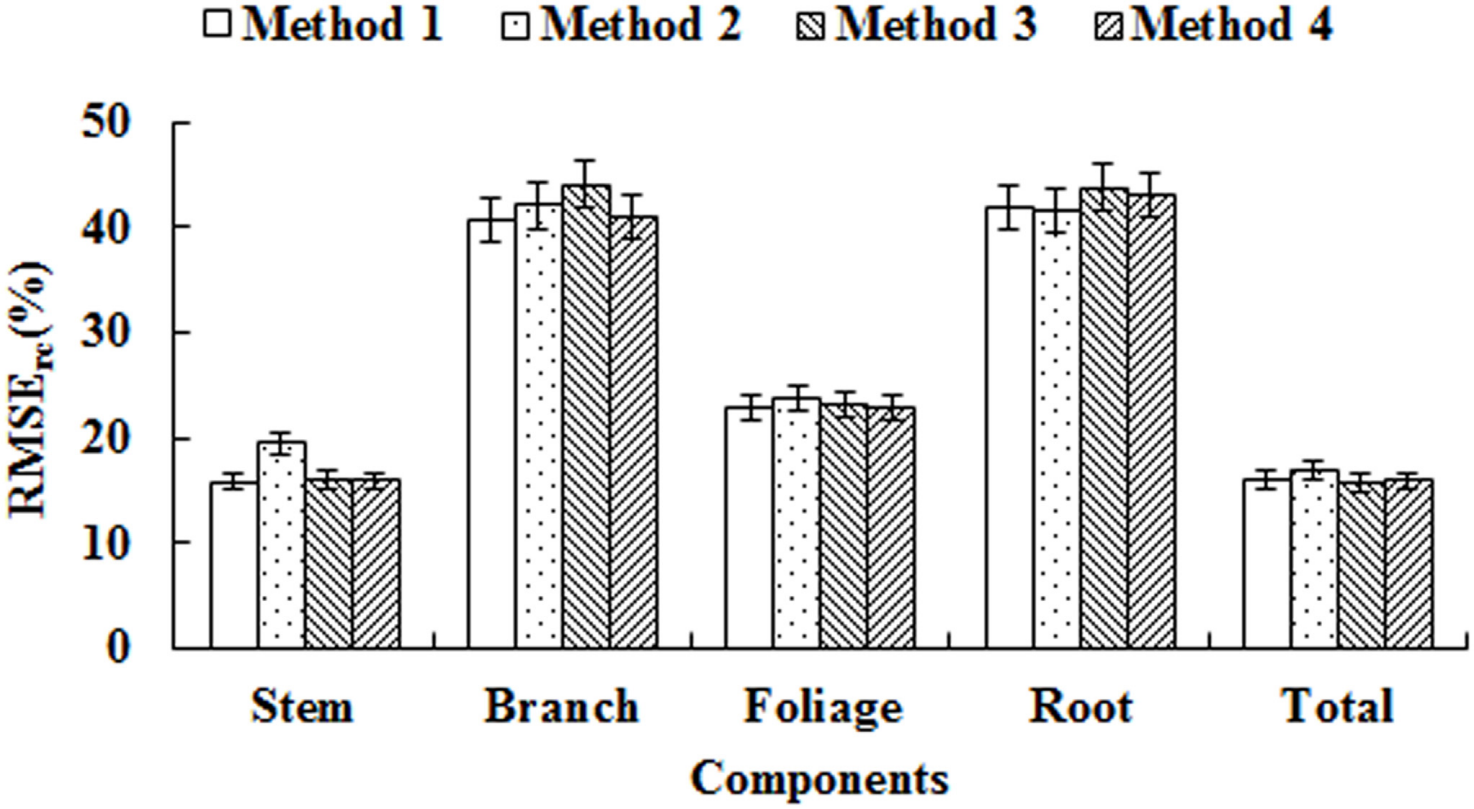
RMAE_rc_ of four methods for the carbon stock prediction of tree components and total. The error bars correspond to the lower and upper limits of the 95% confidence intervals.

Another statistic, namely, relative root mean squared error for carbon (RMSE_rc_) was derived to compare the carbon stock prediction of four methods [9, 16, 18, 40]. The RMSE_rc_ of all tree components and total with the four methods was showed as Fig 4. For tree stem, Method 1 was the best with the lowest RMSE_rc_, followed by Method 4, Method 3, and Method 2. For tree branch, Method 1 was the best method with the lowest RMSE_rc_, followed by Method 4, Method 2 and Method 3. As for foliage, Method 1 was also the best, followed by Method 4, Method 3 and Method 2. The RMSE_rc_ of Method 1 for root was slightly larger than Method 2, and still lower than Method 3 and Method 4. For the total, Method 1 was notably lower than Method 2 but slightly larger than Method 3 and Method 4.

To test the differences among the four methods, the prediction errors were used as the response variable using analysis of variance (ANOVA) with least significant difference (LSD) for multiple mean comparisons. The test results were showed in Table 6. The F-test in ANOVA was statistically significant (p-value < 0.01) for stem, branch, foliage, root and total tree carbon prediction. The blocking (trees) was also significant, indicating the differences among the sampled trees were also relatively large.

**Table 6:** Comparison of treatment means between four methods of carbon stocks evaluation for each component and total, in which the same letters indicate the difference between treatments are not statistically significant.

Method	Stem		Branch	Foliage	Root	Total	
**Method 1**	A	C	A	A	A		B
**Method 2**		C	A	A	A	A	
**Method 3**	B		A	A	A	A	B
**Method4**	B	C	A	A	A		B

The multiple mean comparison results indicated that (1) for stem, Method 1 was significantly different from Method 2 and Method 3, but there was no difference between Method 3 and Method 4, as well as no difference between Method 1 and Method 4 (Table 6). One the other hand, for branch, foliage, and root, there was no difference between the four methods. For the total, there was no significant difference between Method 2 and Method 3, and no difference among Method 1, Method 3 and Method 4 (Table 6).

## Discussion

To investigate which model is better for fitting the biomass and carbon stock data, tree diameter alone and the combination of diameter and height were used as the predictor variables for predicting the tree component and total biomass and carbon stocks in this study. Some researchers also used other tree variables such as crown width, stand age, wood density, diameter outside bark at the base of the live crown, crown height, and etc., which may performed well in some studies [18, 23, 41–44]. However, given the practical difficulties and costs of obtaining crown measurements, tree diameter and height are the most frequently used predictors in model construction. In our study, adding H into the biomass and carbon stock models only increased the R_a_^2^ of stem and root because the differences of tree height at the same tree diameter contributed more information for the biomass/carbon stocks of stem and root [45] and the estimation errors of other tree components (branch, foliage) were increased consistent with the decreased of R_a_^2^ after adding H into the models (Tables 3, 4 and 5). Therefore, we chose D as the sole predictor variable in this study to develop the biomass and carbon stock models. To ensure the additivity of the tree components and total, the compatible biomass and carbon stock models were constructed with the total tree biomass and carbon stock as the constraints based on the allomatric equation [32]. Furthermore, the R_a_^2^ of some tree components might be sacrificed to ensure the additivity for the components and total compared to fit each component separately. Given NSUR is more flexible and has been widely accepted by forest modelers [25, 27, 28], we used NSUR as the only parameter estimation method in this study and did not compare NSUR against others parameter estimation methods.

Currently, the carbon stock calculation is based on either observed carbon concentrations [46] or a commonly accepted carbon conversion factor 0.5, which is multiplied by the estimated tree biomass [47]. However, the prediction error using indirect method is lack of evaluation. In this study we used RMAE_rc_ to compare and evaluate carbon stocks of tree components of individual trees for the indirect method. The statistical tests (ANOVA with LSD) indicated that, for stem, the indirect method using the carbon conversion factor 0.5 produced significantly larger errors than the other two methods. Consequently, the larger prediction error for stem would surely lead to the large prediction error for the total biomass because it is known that more than 50% of the total biomass is located in the stem [16]. It seemed that the other two indirect methods, using either the average carbon concentration of total tree (0.48) (Method 3) or the average carbon concentration of each tree component (stem 0.47, branch 0.48, foliage 0.49, and root 0.48) (Method 4), were superior to the carbon conversion factor 0.5 (Method 2). Although the differences between Method 3 and Methods 4 was not significant, Method 4 would require considerably more time and effort to collect in the field. Thus, Method 3 (the average carbon concentration of total tree) should be sufficient to obtain good carbon stock prediction with the estimated tree biomass from the models. For branch, foliage and root, the prediction error among the four methods was not significant, this can be explained that the real carbon concentration of the three components are more close to the total tree concentration. Lehtonen et al. (2007) [48] analyzed the prediction error of biomass expansion factors and the error from variable measurements and regression functions. Their results showed that most of the error was due to the regression models. Therefore, it is crucial to develop and select the “best” biomass models as the foundation for computing carbon stocks using indirect methods. In our study, we used diameter at breast height and the combination of diameter at breast height and tree height to establish the biomass and carbon stock models. Based on goodness-of-fit and validation results for the different kinds of models, we selected the model which employed diameter at the breast height to make comparison among the four methods to decrease the error of indirect methods deriving from the biomass model as large as possible. There were no significant differences between Method 3 and Method 4 according to the total prediction error estimation indicating that using observed average carbon concentration of individual tree and / or tree components would lead to better prediction for carbon stocks. We concluded that it was not necessary to measure the carbon concentrations for tree components because they are time-consuming and costly. The average carbon concentration of individual tree should be sufficient to obtain accurate computation for carbon stocks given reliable and accurate estimation of biomass.

The results of our study were different from the study of [16], in which they used three methods: 1) estimated total biomass was multiplied by weighted mean carbon concentration; 2) estimated tree compartment biomass was multiplied by average carbon concentration of tree components; and 3) developing carbon stocks prediction model directly. Mello et al. (2012) [16] found that there was no statistical difference among the three methods. However, there were only 30 sample trees included in Mello et al. (2012) [16]. The small sample size might lead to an insignificant comparison. In addition, the biomass and carbon models developed in Mello et al. (2012) [16] did not hold the additivity or compatibility of tree components and total. In addition, the differences of prediction errors among the three methods at tree component levels were not clear in the study of Mello et al. (2012) [16].

## Conclusion

Four methods were used to predict carbon stocks of tree components and total for Korean pine trees in the plantations of northeastern China. Based on the results of goodness-of-fit and model performance statistics of the biomass and carbon stock models, we chose the compatible systems of models with tree diameter as the sole independent variable in this study. The NSUR method was used to estimate the parameters in these models. The prediction errors of four methods for estimating carbon stocks of tree components and total were compared and tested.

The direct method using the compatible carbon stocks models (Method 1) was the best method for predicting the carbon stocks of stem and total. The indirect method using the average carbon concentration of tree (Method 3) and average carbon conversion of each tree component (Method 4) produced relative small prediction errors, while the indirect method using the carbon conversion factor 0.5 (Method 2) was the worse for estimating the carbon stocks for tree components and total of Korean pine in this study. However, there were no significant difference between Method 3 and Method 4. Thus, the average carbon concentration of individual tree rather than the average carbon concentration for each tree component is good enough to calculate the carbon stocks for tree components and total.

## Appendix

The variable allomatric equation takes the form of:

$$\text{Y}={\text{aX}}^{\text{b}}$$ (A1)

If the response variable Y is tree biomass and the independent variable X is tree diameter at breast height D, Eq [A1] becomes:

$$\text{W}={\text{aD}}^{\text{b}}$$ (A2)

In this study, our additive systems of tree biomass were formulated based on the methods by Tang and Li [32], in which the total tree biomass was used as the constraint as follows:

$${\text{W}}_{\text{t}}={\text{ W}}_{\text{s}}+{\text{ W}}_{\text{b}}+{\text{ W}}_{\text{f}}+{\text{ W}}_{\text{r}}$$ (A3)

where W_s_, W_b_, W_f_, W_r_ and W_t_ are the biomass of stem, branch, foliage, root and total, respectively.

Thus, based on Eq [A2], the additive system of tree biomass is as follows:

$$\{\begin{array}{c} {\text{W}}_{\text{s}}={\text{a}}_{\text{1}}{\text{D}}^{{\text{b}}_{\text{1}}}\\ {\text{W}}_{\text{b}}={\text{a}}_{\text{2}}{\text{D}}^{{\text{b}}_{\text{2}}}\\ {\text{W}}_{\text{f}}={\text{a}}_{\text{3}}{\text{D}}^{{\text{b}}_{\text{3}}}\\ {\text{W}}_{\text{r}}={\text{a}}_{\text{4}}{\text{D}}^{{\text{b}}_{\text{4}}}\\ {\text{W}}_{\text{t}}={\text{W}}_{\text{s}}+{\text{W}}_{\text{b}}+{\text{W}}_{\text{f}}+{\text{W}}_{\text{r}}={\text{a}}_{\text{0}}{\text{D}}^{{\text{b}}_{\text{0}}} \end{array}$$ (A4)

With the constraint of total biomass, the biomass of each tree component is further formulated as follows:

$$\left\{ \begin{array}{l} {\text{W}}_{\text{s}}=\frac{{\text{a}}_{\text{1}}{\text{D}}^{{\text{b}}_{\text{1}}}}{{\text{a}}_{\text{1}}{\text{D}}^{{\text{b}}_{\text{1}}}+{\text{a}}_{\text{2}}{\text{D}}^{{\text{b}}_{\text{2}}}+{\text{a}}_{\text{3}}{\text{D}}^{{\text{b}}_{\text{3}}}+{\text{a}}_{\text{4}}{\text{D}}^{{\text{b}}_{\text{4}}}}\cdot {\text{a}}_{\text{0}}{\text{D}}^{{\text{b}}_{\text{0}}}\\ {\text{W}}_{\text{b}}=\frac{{\text{a}}_{\text{2}}{\text{D}}^{{\text{b}}_{\text{2}}}}{{\text{a}}_{\text{1}}{\text{D}}^{{\text{b}}_{\text{1}}}+{\text{a}}_{\text{2}}{\text{D}}^{{\text{b}}_{\text{2}}}+{\text{a}}_{\text{3}}{\text{D}}^{{\text{b}}_{\text{3}}}+{\text{a}}_{\text{4}}{\text{D}}^{{\text{b}}_{\text{4}}}}\cdot {\text{a}}_{\text{0}}{\text{D}}^{{\text{b}}_{\text{0}}}\\ {\text{W}}_{\text{f}}=\frac{{\text{a}}_{\text{3}}{\text{D}}^{{\text{b}}_{\text{3}}}}{{\text{a}}_{\text{1}}{\text{D}}^{{\text{b}}_{\text{1}}}+{\text{a}}_{\text{2}}{\text{D}}^{{\text{b}}_{\text{2}}}+{\text{a}}_{\text{3}}{\text{D}}^{{\text{b}}_{\text{3}}}+{\text{a}}_{\text{4}}{\text{D}}^{{\text{b}}_{\text{4}}}}\cdot {\text{a}}_{\text{0}}{\text{D}}^{{\text{b}}_{\text{0}}}\\ {\text{W}}_{\text{r}}=\frac{{\text{a}}_{\text{4}}{\text{D}}^{{\text{b}}_{\text{4}}}}{{\text{a}}_{\text{1}}{\text{D}}^{{\text{b}}_{\text{1}}}+{\text{a}}_{\text{2}}{\text{D}}^{{\text{b}}_{\text{2}}}+{\text{a}}_{\text{3}}{\text{D}}^{{\text{b}}_{\text{3}}}+{\text{a}}_{\text{4}}{\text{D}}^{{\text{b}}_{\text{4}}}}\cdot {\text{a}}_{\text{0}}{\text{D}}^{{\text{b}}_{\text{0}}} \end{array}\right.$$ (A5)

Dividing a_1_D^b1^,a_2_D^b2^,a_3_D^b3^,a_4_D^b4^, respectively, to the numerator and denominator of each component equation in Eq [A5]. To simplify equation, we define: r_1_ = α_2_/α_1_, r_2_ = α_3_/α_1_, r_3_ = α_4_/α_1_, k_1_ = b_2_−b_1_, k_2_ = b_3_−b_1_, k_3_ = b_4_−b_1_. Thus, we obtain the following equation system:

$$\left\{ \begin{array}{l} {\text{W}}_{\text{s}}=\frac{{\text{a}}_{\text{0}}{\text{D}}^{{\text{b}}_{\text{0}}}}{\text{1}+\frac{{\text{a}}_{\text{2}}}{{\text{a}}_{\text{1}}}\cdot {\text{D}}^{{\text{b}}_{\text{2}}-{\text{b}}_{\text{1}}}+\frac{{\text{a}}_{\text{3}}}{{\text{a}}_{\text{1}}}\cdot {\text{D}}^{{\text{b}}_{\text{3}}-{\text{b}}_{\text{1}}}+\frac{{\text{a}}_{\text{4}}}{{\text{a}}_{\text{1}}}\cdot {\text{D}}^{{\text{b}}_{\text{4}}-{\text{b}}_{\text{1}}}}\\ {\text{W}}_{\text{b}}=\frac{{\text{a}}_{\text{0}}{\text{D}}^{{\text{b}}_{\text{0}}}}{\frac{{\text{a}}_{\text{1}}}{{\text{a}}_{\text{2}}}\cdot {\text{D}}^{{\text{b}}_{\text{1}}-{\text{b}}_{\text{2}}}+\text{1}+\frac{{\text{a}}_{\text{3}}}{{\text{a}}_{\text{2}}}\cdot {\text{D}}^{{\text{b}}_{\text{3}}-{\text{b}}_{\text{2}}}+\frac{{\text{a}}_{\text{4}}}{{\text{a}}_{\text{2}}}\cdot {\text{D}}^{{\text{b}}_{\text{4}}-{\text{b}}_{\text{2}}}}\\ {\text{W}}_{\text{f}}=\frac{{\text{a}}_{\text{0}}{\text{D}}^{{\text{b}}_{\text{0}}}}{\frac{{\text{a}}_{\text{1}}}{{\text{a}}_{\text{3}}}\cdot {\text{D}}^{{\text{b}}_{\text{1}}-{\text{b}}_{\text{3}}}+\frac{{\text{a}}_{\text{2}}}{{\text{a}}_{\text{3}}}\cdot {\text{D}}^{{\text{b}}_{\text{2}}-{\text{b}}_{\text{3}}}+\text{1}+\frac{{\text{a}}_{\text{4}}}{{\text{a}}_{\text{3}}}\cdot {\text{D}}^{{\text{b}}_{\text{4}}-{\text{b}}_{\text{3}}}}\\ {\text{W}}_{\text{r}}=\frac{{\text{a}}_{\text{0}}{\text{D}}^{{\text{b}}_{\text{0}}}}{\frac{{\text{a}}_{\text{1}}}{{\text{a}}_{\text{4}}}\cdot {\text{D}}^{{\text{b}}_{\text{1}}-{\text{b}}_{\text{4}}}+\frac{{\text{a}}_{\text{2}}}{{\text{a}}_{\text{4}}}\cdot {\text{D}}^{{\text{b}}_{\text{2}}-{\text{b}}_{\text{4}}}+\frac{{\text{a}}_{\text{3}}}{{\text{a}}_{\text{4}}}\cdot {\text{D}}^{{\text{b}}_{\text{3}}-{\text{b}}_{\text{4}}}+\text{1}} \end{array}\right.=\left\{ \begin{array}{l} {\text{W}}_{\text{s}}=\frac{{\text{a}}_{\text{0}}{\text{D}}^{{\text{b}}_{\text{0}}}}{\text{1}+{\text{r}}_{\text{1}}{\text{D}}^{{\text{k}}_{\text{1}}}+{\text{r}}_{\text{2}}{\text{D}}^{{}^{{\text{k}}_{\text{2}}}}+{\text{r}}_{\text{3}}{\text{D}}^{{\text{k}}_{\text{3}}}}\\ {\text{W}}_{\text{b}}=\frac{{\text{a}}_{\text{0}}\cdot {\text{r}}_{\text{1}}\cdot {\text{D}}^{{\text{b}}_{\text{0}}+{\text{k}}_{\text{1}}}}{\text{1}+{\text{r}}_{\text{1}}{\text{D}}^{{\text{k}}_{\text{1}}}+{\text{r}}_{\text{2}}{\text{D}}^{{}^{{\text{k}}_{\text{2}}}}+{\text{r}}_{\text{3}}{\text{D}}^{{\text{k}}_{\text{3}}}}\\ {\text{W}}_{\text{f}}=\frac{{\text{a}}_{\text{0}}\cdot {\text{r}}_{\text{2}}\cdot {\text{D}}^{{\text{b}}_{\text{0}}+{\text{k}}_{\text{2}}}}{\text{1}+{\text{r}}_{\text{1}}{\text{D}}^{{\text{k}}_{\text{1}}}+{\text{r}}_{\text{2}}{\text{D}}^{{}^{{\text{k}}_{\text{2}}}}+{\text{r}}_{\text{3}}{\text{D}}^{{\text{k}}_{\text{3}}}}\\ {\text{W}}_{\text{r}}=\frac{{\text{a}}_{\text{0}}\cdot {\text{r}}_{\text{3}}\cdot {\text{D}}^{{\text{b}}_{\text{0}}+{\text{k}}_{\text{3}}}}{\text{1}+{\text{r}}_{\text{1}}{\text{D}}^{{\text{k}}_{\text{1}}}+{\text{r}}_{\text{2}}{\text{D}}^{{}^{{\text{k}}_{\text{2}}}}+{\text{r}}_{\text{3}}{\text{D}}^{{\text{k}}_{\text{3}}}} \end{array}\right.$$ (A6)

Adding tree total height H to Eq [A2] results in another popular biomass model:

$$\text{Y}={\text{a(D}}^{\text{2}}{\text{H)}}^{\text{b}}$$ (A7)

Following the same derivation process above, the compatible biomass and carbon stocks models can be constructed as follows:

$$\left\{ \begin{array}{l} {\text{W}}_{\text{s}}={\text{a}}_{\text{0}}{\text{D}}^{{\text{2b}}_{\text{0}}}{\text{H}}^{{\text{b}}_{\text{0}}}\text{/(1}+{\text{r}}_{\text{1}}{\text{D}}^{{\text{2k}}_{\text{1}}}{\text{H}}^{{\text{k}}_{\text{1}}}+{\text{r}}_{\text{2}}{\text{D}}^{{\text{2k}}_{\text{2}}}{\text{H}}^{{\text{k}}_{\text{2}}}+{\text{r}}_{\text{3}}{\text{D}}^{{\text{2k}}_{\text{3}}}{\text{H}}^{{\text{k}}_{\text{3}}})\\ {\text{W}}_{\text{b}}={\text{a}}_{\text{0}}{\text{r}}_{\text{1}}{\text{D}}^{{\text{2k}}_{\text{1}}+{\text{2b}}_{\text{0}}}{\text{H}}^{{\text{k}}_{\text{1}}+{\text{b}}_{\text{0}}}\text{/(1}+{\text{r}}_{\text{1}}{\text{D}}^{{\text{2k}}_{\text{1}}}{\text{H}}^{{\text{k}}_{\text{1}}}+{\text{r}}_{\text{2}}{\text{D}}^{{\text{2k}}_{\text{2}}}{\text{H}}^{{\text{k}}_{\text{2}}}+{\text{r}}_{\text{3}}{\text{D}}^{{\text{2k}}_{\text{3}}}{\text{H}}^{{\text{k}}_{\text{3}}})\\ {\text{W}}_{\text{f}}={\text{a}}_{\text{0}}{\text{r}}_{\text{2}}{\text{D}}^{{\text{2k}}_{\text{2}}+{\text{2b}}_{\text{0}}}{\text{H}}^{{\text{k}}_{\text{2}}+{\text{b}}_{\text{0}}}\text{/(1}+{\text{r}}_{\text{1}}{\text{D}}^{{\text{2k}}_{\text{1}}}{\text{H}}^{{\text{k}}_{\text{1}}}+{\text{r}}_{\text{2}}{\text{D}}^{{\text{2k}}_{\text{2}}}{\text{H}}^{{\text{k}}_{\text{2}}}+{\text{r}}_{\text{3}}{\text{D}}^{{\text{2k}}_{\text{3}}}{\text{H}}^{{\text{k}}_{\text{3}}})\\ {\text{w}}_{\text{r}}={\text{a}}_{\text{0}}{\text{r}}_{\text{3}}{\text{D}}^{{\text{2k}}_{\text{3}}+{\text{2b}}_{\text{0}}}{\text{H}}^{{\text{k}}_{\text{3}}+{\text{b}}_{\text{0}}}\text{/(1}+{\text{r}}_{\text{1}}{\text{D}}^{{\text{2k}}_{\text{1}}}{\text{H}}^{{\text{k}}_{\text{1}}}+{\text{r}}_{\text{2}}{\text{D}}^{{\text{2k}}_{\text{2}}}{\text{H}}^{{\text{k}}_{\text{2}}}+{\text{r}}_{\text{3}}{\text{D}}^{{\text{2k}}_{\text{3}}}{\text{H}}^{{\text{k}}_{\text{3}}}) \end{array}\right.$$ (A8)

where the model coefficients a_0_, b_0_, r_1_, r_2_, r_3_, k_1_, k_2_ and k_3_ are estimated from the tree biomass.

## Supporting Information

S1 File Biomass data used in the study. (XLS)
